# Identification of a common Ara h 3 epitope recognized by both the capture and the detection monoclonal antibodies in an ELISA detection kit

**DOI:** 10.1371/journal.pone.0182935

**Published:** 2017-08-11

**Authors:** Lipei Zhao, Liang Zhao, Buchang Zhang, Jason M. Robotham, Kenneth H. Roux, Hengli Tang

**Affiliations:** 1 Institute of Health Sciences, Anhui University, Hefei, Anhui, PR China; 2 Department of Biological Science, Florida State University, Tallahassee, Florida, United States of America; International Nutrition Inc, UNITED STATES

## Abstract

Allergy to peanuts has become a common and severe problem, especially in westernized countries. In this study, we evaluated the target and epitope specificity of the capture and detection mouse monoclonal antibodies (mAbs) used in a commercial peanut allergen detection platform. We first identified the target of these antibodies as Ara h 3 and then used an overlapping peptide array of Ara h 3 to determine the antibody-binding epitopes. Further amino acids critical for the binding via alanine substitutions at individual amino acid residues within the epitope were mapped. Finally, inhibition ELISA and inhibition immunoblotting using a recombinant Ara h 3 protein were performed to confirm these results. Surprisingly, the capture and detection mAbs showed identical binding characteristics and were presumed to represent two isolates of the same clone, a notion supported by both isoelectric focusing electrophoresis and Liquid chromatography–mass spectrometry experiments. The simultaneous binding of a pair of identical mAbs to an individual allergen such as Ara h3 is attributed to the multivalency of the analyte and has implications for developing diagnostic assays for additional multimeric allergens.

## Introduction

Peanut (*Arachis hypogaea* L.) is one of the most common inducers of type I IgE-mediated food allergy [1, 2]. The occurrence of peanut hypersensitivity seems to be increasing [3–5] and many peanut allergens have been identified [6–13]. Peanut allergy is mediated by the production of IgE antibody and largely due to epitopes composed of linear amino acid sequences that don’t contain carbohydrates [11, 14–16]. In contrast to milk and egg allergy, peanut allergy often persists through adulthood [11, 17, 18]. Although difficult, the best approach remains a complete avoidance by allergic individuals of the offending allergens [2, 15, 19–21] because immunotherapy for peanut allergy is not yet available [22]. Over the past decades, several peanut allergen proteins have been characterized [11, 23–27] with corresponding immune-detection methods developed [28–30] In addition, commercial platforms for detecting potentially unwanted peanut antigens in foods have been developed. Their widespread application in the food industry and regulatory bodies has helped reduce serious allergic reactions caused by unintentional consumption in sensitive individuals. The recent development of a monoclonal antibody (mAb)-based commercial peanut testing kit (MonoTrace^™^ Peanut ELISA Kit, BioFront Technologies, Tallahassee, FL, USA) has afforded a unique opportunity to investigate the nature of the targeted protein and constituent epitopes with a consistent and renewable antibody supply because of the monoclonal nature of the antibodies used. We found that both the capture and detection mAbs used in the kit’s sandwich ELISA assay recognize the major allergen, Ara h 3, and target the same epitope. Ara h 3 is an 11S globulin seed storage protein. Structurally, it is in the form of a homohexamer wherein two ring trimers are bound face-to-face [31, 32]. As such, it is perhaps not surprising that two mAbs clones targeting the identical epitope could function in a sandwich ELISA format. In addition, we show that the two mAbs are likely identical and produced by two independent clones generated in vitro for the same hybridoma.

## Material and methods

### Peanut sample, extraction buffer and mAbs

Peanut flour, extraction buffer and mAbs (P1 clone: 4E7, P2 clone: 5C5, as well as P5 clone) of peanut and W1 clone of walnut were provided by BioFront Technologies Inc. (Tallahassee, FL USA). The mAbs used in the kit were selected using a direct-binding ELISA (as described for almond, [33]) on culture supernatants from the clones derived from a hybridoma fusion using the spleen from a single mouse immunized with peanut extract.

### Extraction of peanut proteins

Peanut flour and 1 X extraction buffer were mixed at a ratio of 1:20 and incubated at 62°C for 10 min with shaking at 2 min intervals. The aqueous peanut protein extract was centrifuged at 3000 x g for 10 min with saving of the supernatant. The protein concentration was determined using the Coomassie Plus^™^ (Bradford) Assay Kit (Thermo Scientific, Waltham, MA, USA).

### Electrophoresis and immunoblotting

For SDS-PAGE analysis of peanut allergen, flour samples were boiled for 10 min in reducing or non-reducing buffer, and electrophoresed at 80 V in stacking gels, and 120 V in separating gels (Mini-PROTEIN^®^ Tetra Cell Systems and PowerPac HC power supply, BioRad, Hercules, CA, USA). To ensure adequate protein separation, gels were stained with Coomassie brilliant blue R-250 (BBI Life Sciences, Markham, Ontario, Canada) according to manufacturer’s instructions.

Proteins were electrophoretically transferred to 0.22 μm polyvinylidene fluoride (PVDF) membranes (BioRad) in a transblot apparatus (BioRad). Ponceau S staining was done to visualize the transferred proteins. The PVDF membranes were blocked for 1 h at room temperature (RT) in PBS/5% nonfat dry milk (NFDM, BBI Life Sciences, Markham, Ontario, Canada)/0.2% Tween 20 to prevent non-specific protein binding. The PVDF blots were then rinsed for 2 min with PBS-T (PBS/0.2% Tween 20) and incubated with the diluted mAbs in the blocking buffer overnight at 4°C with rocking. The strips were then washed 3X for 10 min each wash in PBS-T and incubated at RT for 1 h with HRP-conjugated goat anti-mouse IgG (Santa Cruz Biotechnology, Dallas, TX, USA) in blocking buffer (PBS-T/5% NFDM). The strips were then washed 3X for 10 min each wash and incubated with ECL Western substrate (GE Healthcare, Chicago, Il, USA). The signals were then visualized by a chemical imaging system (5500 Multi, Tanon, Shanghai, China).

### Solid-phase peptide (SPOTs) synthesis

On the basis of the published amino acid sequence of Ara h 3 (Genbank accession number AF093541), 167 overlapping 13-amino acid peptides, each offset by 3 amino acids, were synthesized; these corresponded to the entire amino acid length of the Ara h 3 large and small subunits. Twelve peptides synthesized as variations of the target peptide (EYEYDEEDRRRG) were created through single-site alanine substitutions at each position along the amino acid sequence.

Peptides were synthesized on derivatized cellulose membranes embedding free hydroxyl groups using fluorenlymethoxy carbonyl-derived (Fmoc) amino acids according to manufacturer’s (Pepnoch Biotech Inc., Beijing, China) instructions. Membranes containing synthetic peptides were probed immediately with the mAbs.

### IgG binding to solid-phase synthetic peptides (SPOTs-analysis)

The membranes containing peptides were activated by washing 3X in absolute ethanol for 10 min and equilibrated in TBS-T (TBS containing 0.2% Tween 20) 3X for 10 min each. The membranes were then incubated for 4 h at RT or overnight at 4°C in blocking buffer as directed by the manufacturer (Pepnoch Biotech Inc.). Membranes were then washed in TBS-T for 5 min and incubated overnight at 4°C or more than 2 h at RT with mAbs diluted in blocking buffer. This incubation was followed by three 10-min washes in TBS-T and an incubation with HRP-conjugated goat anti-mouse IgG (Santa Cruz Biotechnology, Dallas, TX, USA) diluted 1:5,000 in a mixture of TBS, 5% sucrose, 4% NFDM, and 0.2% Tween 20. The membranes were finally washed for 3X for 10 min each in in TBS-T. IgG-peptide reactivity was identified by exposure to Kodak X-OMAT x-ray film (Eastman Kodak, Rochester, NY, USA).

### ELISA Inhibition

Peanut protein extracts were diluted to a final concentration of 1 μg/ml in coating buffer (0.1 M of sodium carbonate-bicarbonate buffer, pH 9.6) and 100 μl aliquots were transferred to wells of a high affinity protein-binding 96-well microtiter plate (NEST Biotechnology, Wuxi, Jiangsu, China) for incubation overnight at RT. The plate was washed 5X with rinse buffer (PBS-T, pH 7.4), blocked in 1% BSA in PBS-T overnight at RT, and then washed 3X with PBS-T. One hundred microliters of each blocking mAbs (P1, P2, P5, and W1) were then added to appropriate wells and incubated for 2 h at RT. After five washes with PBS-T, the plates were incubated for 30 min at RT with HRP-conjugated P1, diluted 1:20,000 in blocking buffer. The plates were again washed with PBS-T, following by the addition of TMB substrate (TransGen Biotech Inc., Beijing, China) and incubation for 25 min at RT. The reactions were stopped by the addition of 100 μl of 0.5 M H_2_SO_4_ to each well, and absorbance was read at 450 nm using a microplate reader (SpectraMAX 190, Molecular Devices, Shanghai, China). All assays were performed in triplicate.

### PCR amplification, cloning, and sequencing

For recombinant rAra h 3 production, we synthesized a DNA fragment corresponding to the codon-optimized Ara h 3 cDNA and designed the appropriate PCR primers (General Biosystems Inc., Chuzhou, Anhui, China) that matched the 5’ and 3’ end of the cDNA. In addition, the primers incorporated a *NheI* site at the 5’ end and a *SalI* site at the 3’ end of the Ara h 3 sequence. The sequences of the primers were 5’- TATGGCTAGCTTTCGTCAGC-3’ (H3-5’primer) and 5’-CCGTCGACTGCAACGGCAG-3’ (H3-3’primer). The Ara h 3 cDNA was amplified by PCR and cloned into the *NheI/ SalI* restriction sites of the pET-24(b)^+^ vector (Novagen, Billerica, Massachusetts). The expression vector contains the kanamycin resistance gene and can encode sequence for a 6xHis tag at the C-terminus of the rAra h 3 protein. The recombinant plasmid was transformed into competent *Escherichia coli* DH5α cells and incubated overnight at 37°C. Positive clones were identified by PCR and confirmed by sequencing.

### Bacterial expression and purification of recombinant Ara h 3

Induction of recombinant protein expression was carried out by adding isopropyl-B-D-thiogalactopyranoside (IPTG) to a final concentration of 0.8 mM to the bacterial culture of BL21 (DE3) when the optical density of the culture reached of absorbance of A_600_ = 0.8. Cell extracts suspended in SDS sample buffer was boiled briefly before loading onto a SDS-PAGE gel. Recombinant protein was stained with Coomassie brilliant blue.

Purification of recombinant protein from bacterial lysates was performed under denaturing condition. Cell extracts in column binding buffer (5 mM imidazole, 20 mM Tris-HCl, 0.5 M NaCl, and 8 M urea) were incubated with sonication-sheared DNA on ice for 30 min and subjected to centrifugation at 4°C at 12,000 rpm for 30 min, which removed unbroken cells and cell debris. The supernatant was cleared through a 0.45-μm filter and then loaded onto the His-Select Nickel Affinity gel (Sigma-Aldrich) column, which had been pre-washed with ddH_2_O, treated with 0.1 M NiSO_4_ to charge the column, and equilibrated with 10 volumes of cold binding buffer. Unbound proteins were washed with 10 volumes of binding buffer and the recombinant protein was eluted using 5 volumes of elution buffer (50 mM imidazole, 20 mM Tris-HCl, 0.5 M NaCl, and 8 M urea), following by a final wash with higher imidazole (100 mM imidazole/500 mM imidazole, 20 mM Tris-HCl, 0.5 M NaCl, and 8 M urea). The eluted rAra h 3 recombinant protein was dialyzed at 4°C in 20 mM Tris-HCl, 0.5 M NaCl buffer overnight with one change of buffer. We then stored the purified recombinant protein at either 4°C (briefly storage) or frozen at –80°C for later use.

### Gel electrophoresis (SDS-PAGE) and inhibition immunoblotting

Total peanut protein extract samples (500 ng/well) were boiled in reducing and non-reducing sample buffers and then subjected to SDS-PAGE. Immunoblots were rinsed for 2 min in PBS-T, blocked for 1 h at RT in blocking buffer and rinsed again. For inhibition experiments, 100 μl (1.85 μg) and 200 μl aliquots of rAra h 3 and 100 μl of storage buffer (20 mM Tris-HCl, 0.5 M NaCl) (control) were preincubated with 0.66 μl mAb P1 overnight at 4°C. Afterwards, PVDF strips containing blotted peanut protein extract (and control strips) were blocked. The mixtures were then incubated with the strips for 2 h at RT with rocking. In addition, the same concentrations of P1 mAb were incubated with PVDF strips containing blotted peanut extract for 2 h at RT. Three final 10-min washes were done with PBS-T, and the strips incubated with the appropriate HRP-labeled second antibody. Reactive bands were identified by exposure to Kodak X-OMAT x-ray film (Eastman Kodak).

### Capillary isoelectric focusing electrophoresis

The samples were focused with the CE instrument (PA800 plus, Beckman Coulter, Inc., Brea, CA, USA) using an ampholyte pI gradient 3 to 10, supplied with the Capillary Isoelectric Focusing kit (Beckman Coulter, Inc.). Three microliters of samples were applied, and an electric current of 15 V was applied for 6 min and then increased to 21 V for 45 min. Air pressure was increased from 0.5 to 2 psi during this time period. The cIEF was performed according to the manufacture’s manual.

### Protein modeling

The crystallographic structure of hexameric Aha h 3 (PDB 3C3V)[32] was visualized and modified using Pymol molecular graphics program (http://pymol.org/).

## Results

### P1 and P2 mAbs bind a single antigen in the peanut extract

**Fig 1:**
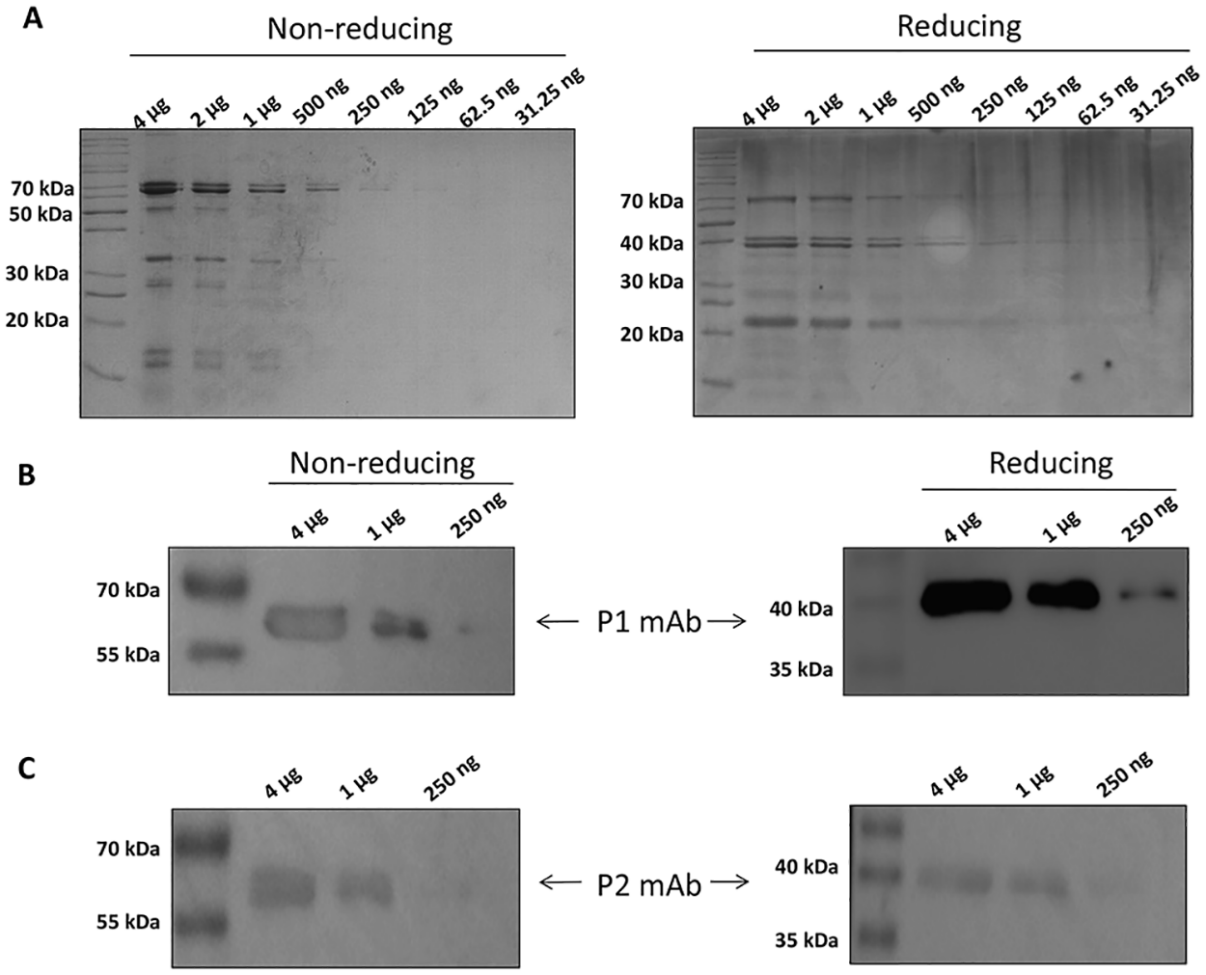
P1 and P2 recognize a single antigen in peanut extract. A. SDS-PAGE analysis of peanut protein extract stained with Coomassie brilliant blue R-250 in non-reducing and in reducing conditions. B-C. Immunoblot of peanut protein extract with P1 (B) or P2 (C) mAb in non-reducing (left) and in reducing (right) conditions.

In this study, we identify the target protein and epitopes of two mAbs incorporated into a commercial peanut allergen detection kit (MonoTrace Peanut ELISA, BioFront Technologies). The kit is designed as a standard sandwich ELISA wherein one antibody, the capture mAb (P1), is bound to the microtiter plate and the other, the enzyme-labeled detection mAb (P2), is added subsequently to bind the captured antigen. Both mAbs were derived from a single mouse previously immunized with peanut extract. We first analyzed the protein profile of peanut extract by SDS-PAGE. As expected, peanut protein extract revealed multiple major protein fractions under non-reducing and reducing conditions (Fig 1A). We then probed the immunoblots with the two mAbs. Both P1 and P2 bound to one band about 60 kDa in non-reducing conditions and one band about 40 kDa in reducing conditions (Fig 1B and 1C). The protein bands detected by these two mAbs had very similar electrophoretic motilities under both conditions. The fact that the epitope is stable under the harsh 100°C reducing conditions with SDS present also suggests that the mAbs bind linear epitopes.

### Identification of Ara h 3 as the antigen target of P1 and P2 mAbs

**Fig 2:**
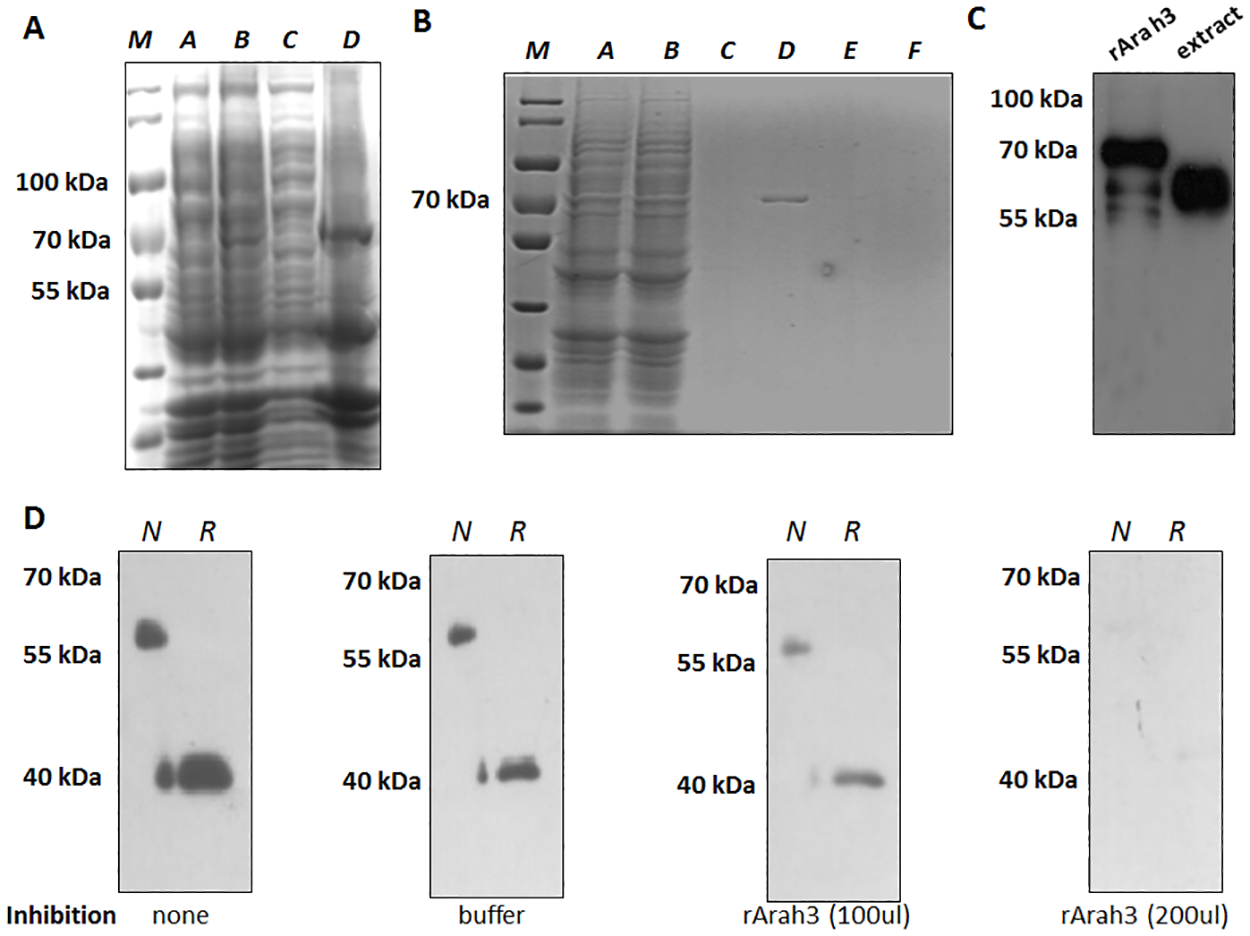
Identification of Ara h 3 as the target antigen of P1 and P2. A. Induction of recombinant Ara h 3 protein expression. Lane *A*, un-induced (vector with DNA insert); Lane *B*, 20-hr induction; Lane *C*, supernatant after ultrasonic homogenization; Lane *D*, precipitation after ultrasonic homogenization. B. Affinity purification of rAra h 3. The recombinant Ara h 3 protein was eluted at 50 mM imidazole concentration visualized through Coomassie blue staining. Lane *A*, post-centrifugation supernatant; Lane *B*, flow-through; Lane *C*, binding buffer eluate; Lane *D*, 50 mM imidazole eluate; Lane E, 100 mM imidazole eluate; Lane *F*, 500 mM imidazole eluate. C. Immuno-blotting of recombinant Ara h 3 and crude peanut extract with P1 mAb. D. Competition of P1 binding to native Ara h 3 in peanut extract by recombinant Ara h 3 protein. N: non-reducing; R: reducing. PVDF blots of total peanut extract were probed with mouse mAb P1 in the absence or the presence of the indicated inhibitors.

The 60 kDa and 40 kDa banding pattern for non-reducing and reducing conditions, respectively, is consistent with the mAbs binding to the 11S globulin Ara h 3 allergenic protein [32]. To confirm that Ara h 3 was the target of P1 and P2, we expressed and purified rAra h 3 and performed immunoblot assays. His-tagged rAra h 3 was expressed in *E*. *coli* and purified to homogeneity by affinity purification (Fig 2A and 2B). Immunoblotting with P1 mAb confirmed that the rAra h 3 exhibited the same strong binding to P1 as the native Ara h 3 protein in the peanut extract, although the additional tag slightly altered the gel mobility of the recombinant protein (Fig 2C). We next demonstrated that the rAra h 3 was antigenically equivalent to the native form by showing that soluble rAra h 3 could inhibit P1 binding to native Ara h 3 present in the peanut extract in a dosage-dependent manner (Fig 2D). Similar results were obtained for the P2 antibody (data not show).

### Epitope mapping of the Ara h 3 P1 and P2 epitopes

**Fig 3:**
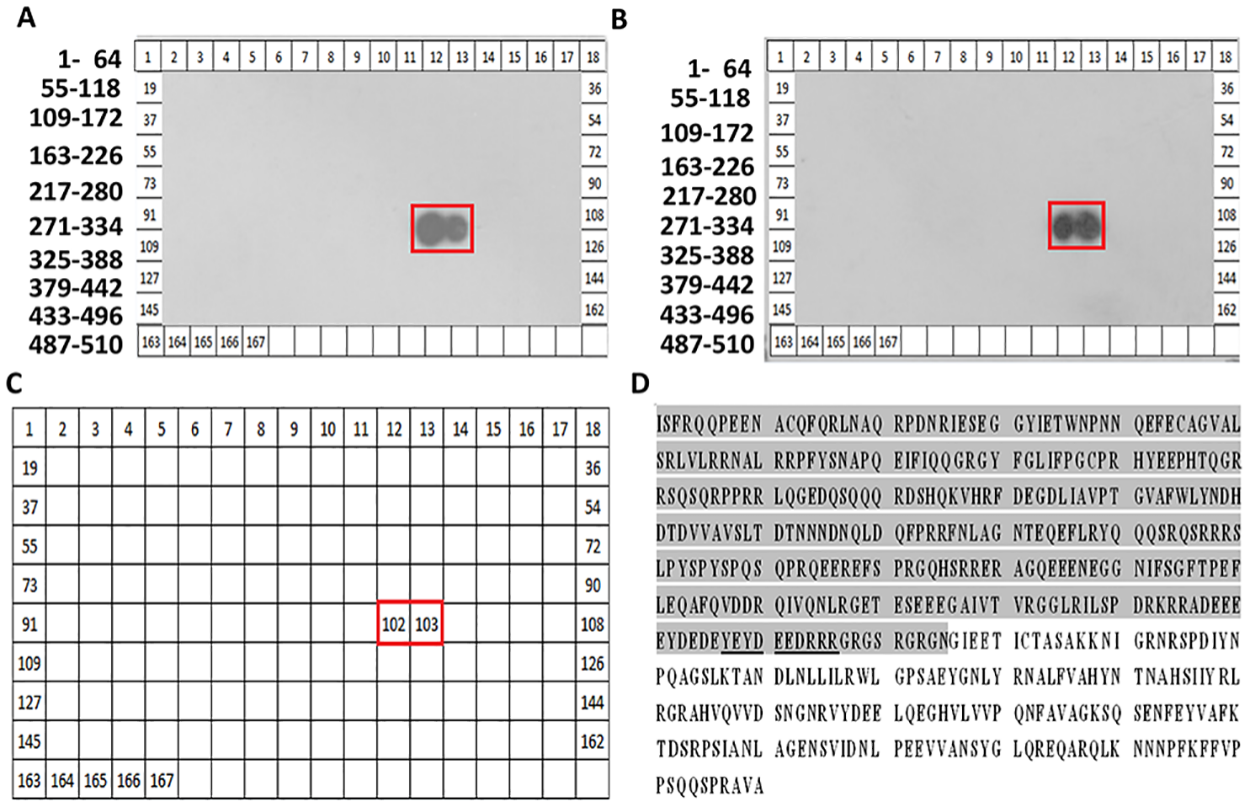
The P1/P2 epitope on the Ara h 3 allergen. The Ara h 3 full length sequence was covered by 167 synthetic peptides of 13-amino acids per peptide, offset from each other by 3 amino acids. These peptides were arranged in an array format and probed with the specific mAbs P1 (A) and P2 (B). The positions of the peptides within the Ara h 3 sequence are shown on the left. C. Peptides at sites 102–103 show recognition with the two mAbs in the membrane. D. The amino acid sequence of the Ara h 3. The shaded areas represent the large subunit of Ara h 3. The positions underlined correspond to the specific peptide targeted as shown in (A) and (B).

Next we mapped the epitope (s) of P1 and P2 on Ara h 3 using spot-based epitope mapping. To this end, 167 overlapping solid phase peptides, constituting the entire length of the Ara h 3 large and small subunits, were synthesized on a filter array and probed with P1 and P2 (Fig 3A–3C). Both mAbs recognized the same two adjacent spots (#102 and #103 on the array), which represent a single linear peptide on the large subunit with a sequence of YEYDEEDRRR (Fig 3D). Interestingly, this sequence partially overlaps with one of the four dominant epitopes bound by human IgE samples isolated from peanut allergic patients.

### Alanine scanning analysis reveals amino acids critical for mAb binding

**Fig 4:**
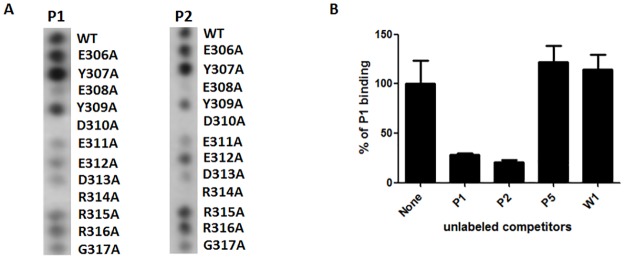
Alanine scanning of the P1/P2 epitope. A. Membranes containing wild-type (WT) E306-G317 peptide and peptides synthesized with single alanine substitutions at each position were probed with the P1 and P2 mAbs. B. Inhibition of HRP- conjugated P1 by unlabeled P1 and P2 in an inhibition ELISA. The competing antibodies are used in 20x excess relative to the labeled P1 antibody. Experiments were performed in triplicates and standard error bars are shown for all measurements.

To map the amino acids of the Ara h 3 epitope that are essential for binding to each mAb, we conducted a mutagenesis study by synthesizing a series of peptides, each with a different single alanine substitution (alanine scanning) to cover each position of the mapped epitope. Fig 4A shows the result of a peptide filter membrane containing both the wild-type and the various mutant epitope peptides probed with the peanut mAbs. The wild-type peptides gave positive signals with both P1 and P2 mAbs as expected. More importantly, the two antibodies showed remarkable similarities in the binding patterns in this alanine scanning peptide array. There was no, or a greatly reduced binding by the mAbs to mutant peptides containing alanine substitutions at positions 308, 310, 311, 313, 314, and 317, demonstrating the critical role of these residues in mediating antibody-binding. Alanine replacement at positions 309, 312, 315, and 316 showed a somewhat reduced binding whereas alanine substitution at position 306 had no effect on binding. Interestingly, an increase in binding signal for both P1 and P2 was observed when the tyrosine at position 307 was replaced by an alanine, suggesting this particular tyrosine residue may have a negative effect on antibody recognition.

The mutagenesis experiment suggested an overlapping spatial relationship between the P1 and P2 epitopes on the Ara h 3 protein. We proceeded to confirm that P2 can indeed compete with P1 for binding to Ara h 3 using an inhibition ELISA assay. Peanut extracts were adsorbed onto a 96-well microtiter plate as antigen for binding. Unconjugated mAbs (competitors) were serially diluted and added to the plate, followed by the addition of HRP-conjugated P1. As expected, unconjugated P1 (positive control) inhibited binding of HRP-conjugated P1 to Ara h 3 in a competitive fashion (Fig 4B). Unconjugated P2 also effectively inhibited HRP-P1 binding, suggesting overlapping or possibly identical epitope recognition. Addition of negative control mAbs W1 and P5 (W1 is a walnut antigen-specific mAb and P5 is another peanut-specific mAb which does not recognize Ara h 3 protein on the western blots) did not result in inhibition of HRP-conjugated P1 binding (Fig 4B).

### 3-D modeling identifies epitope location

**Fig 5:**
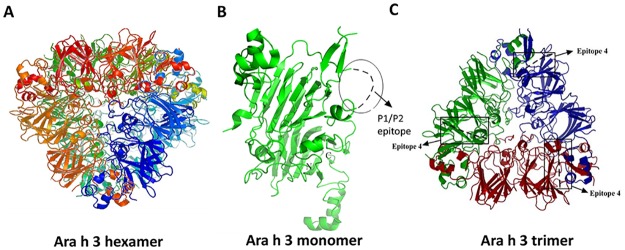
Structures of hexamer, monomer, and trimer of Ara h 3 proteins. A. The hexameric structure of Ara h3 (PDB: 3C3V). B. The P1/P2 epitope is located on an undefined and presumably flexible loop near the C-terminal end of the large subunit of the Ara h 3. C. Mapping of a 3-D structure of Ara h 3 and the P1/P2 epitopes (also named “epitope 4” in Rabjohn et al.) in a trimer (half molecule) model. Each monomeric subunit is colored differently.

Ara h 3 is structurally a member of the cupin superfamily of proteins and composed of six identical subunits, each of which is heterodimeric itself (Fig 5A). The six subunits are arranged in two layers with each layer composed of three subunits oriented radially around a central axis [31]. The available crystal structure of Ara h 3 [32], though nearly complete, does not include the amino acid segment identified in the peptide scanning array. The missing segment is at the surface of the subunit and likely escaped the crystal packing forces due to its location and inherent flexibility (Fig 5B). When mapped to one of the trimeric halves, it appears that the epitope loops project perpendicularly from near the periphery of monomers from the layer (Fig 5C). With this arrangement, it is easy to envision multiple capture and/or detection antibodies binding simultaneously even if they recognize this same epitope on the monomer.

## Discussion

In this study, we demonstrate that the two peanut mAbs, P1 (clone 4E7) and P2 (clone 5C5), that are components of a commercial sandwich ELISA kit, recognize Ara h 3, a major peanut allergen protein that is frequently recognized by IgE in peanut hypersensitive human population. In the ELISA assay, P1 serves as the solid phase capture antibody and P2 as the enzymatically labeled detection antibody.

Despite the ability of these two mAbs to function as a pair in the sandwich format, we obtained multiple lines of experimental evidences indicating that both P1 and P2 mAbs recognize the same linear epitope on the Ara h 3 protein. These included reciprocal inhibitions of epitope-binding by the two antibodies and the alanine-scanning mutagenesis study which showed that the same set of amino acids are critical for binding to both P1 and P2. Because both clones were isolated from the same immunized mouse, at least two hypotheses could explain these results: (1) two independent clones were selected and, by chance, they recognize the same epitope in the same patch (i.e., they interact with the same key residues); or (2) P1 and P2 represent two isolates of the same hybridoma clone (i.e., P1 and P2 are identical antibodies). Both capillary isoelectric focusing electrophoresis (cIEF) and Liquid chromatography–mass spectrometry (LCMS) experiments support the second hypothesis (S1 and S2 Tables). The cIEF experiments demonstrated that the two antibodies have very similar IEF profiles and the LCMS experiments showed identical masses for the peptides generated from enzymatic digestion of the two antibodies. These results strongly support the notion that the original hybridoma clones P1 and P2 secrete the same antibody. Because these two clones were generated from the same mouse immunized with peanut extract, they likely represent two members of a predominant anti- Ara h 3 clone within the mouse that were independently selected during the screening process.

For identical antibodies to function successfully in a sandwich ELISA, the antigen must display at least two solvent accessible cognate epitopes and the epitopes must be positioned in such a way that the binding of one antibody will not sterically hinder the binding of the other. The 11S storage protein Ara h 3 fulfills these requirements as it is a hexamer consisting of six identical subunits [32]. Molecular modeling reveals that the epitopes are in a flexible loop located on the periphery of the two adjacent trimeric rings that comprise the molecule. Consequently at least three and possibly as many as six copies of this same epitope would be available for binding in the fully assembled hexamer. This particular region of 11S globulins has previously been identified as an antibody target in several tree nuts [33, 34] and in peanut where it was recognized by IgE from peanut-allergic patients [11]. In fact, the common epitope for P1 and P2 identified in our study overlaps with the IgE-binding epitope 4 described by Rabjohn et al [11] where they showed that human serum IgE samples from 38% of the peanut hypersensitivity patients previously shown to target rAra h3 also recognized epitope 4. The critical amino acids for antibody-binding mapped by mutagenesis are also by-in-large congruent for the human IgE (Rabjohn et al.) and the mouse IgGs (this study). The independent generation of antibodies against this epitope in human and mouse indicates that murine mAb-based diagnostics can detect clinically relevant allergenic epitopes. And given the common oligomeric nature of food allergen proteins, it would be interesting to determine in future work whether any additional commercial ELISA kits also rely on pairs of identical antibodies for antigen capture and detection. In addition to provide information on potential multimeric nature of the target allergen, a deeper understanding of the availability of single or multiple epitopes in the assay may help optimization efforts to deal with difficult food matrixes or harsh processing conditions that may selectively inactivate epitopes.

## Supporting information

S1 Table Capillary isoelectric focusing electrophoresis results for P1 and P2. (DOCX)

S2 Table Peptide sequences and mass of P1 and P2 obtained from 1D-LCMS experiments. (DOCX)
