# Supplementary material for: Identification of a common Ara h 3 epitope recognized by both the capture and the detection monoclonal antibodies in an ELISA detection kit
Source: PLoS One. 2017 Aug 11;12(8):e0182935. doi: 10.1371/journal.pone.0182935 (PMC5553815; doi:10.1371/journal.pone.0182935)
Supplement: S1 Table — (DOCX) [file pone.0182935.s001.docx]

**S1-Table. Capillary isoelectric focusing electrophoresis results for P1 and P2.**

| Test | P1 | | P2 | |
| --- | --- | --- | --- | --- |
| Experiment 1 | pI:5.86 | pI:6.54 | pI:5.86 | pI:6.54 |
| Experiment 2 | pI:5.83 | pI:6.51 | pI:5.87 | pI:6.56 |
| Average | pI:5.845 | pI:6.525 | pI:5.865 | pI:6.55 |
