# Supplementary material for: Identification of a common Ara h 3 epitope recognized by both the capture and the detection monoclonal antibodies in an ELISA detection kit
Source: PLoS One. 2017 Aug 11;12(8):e0182935. doi: 10.1371/journal.pone.0182935 (PMC5553815; doi:10.1371/journal.pone.0182935)
Supplement: S2 Table — (DOCX) [file pone.0182935.s002.docx]

**S2-Table. Peptide sequences and mass of P1 and P2 obtained from 1D-LCMS experiments.**

| Most abundant peptide | P1 (MH+) | P2 (MH+) |
| --- | --- | --- |
| Peptide 1 | K.APQVYTIPPPK.E (1210.68304) | K.APQVYTIPPPK.E (1210.68304) |
| Peptide 2 | K.DVLTITLTPK.V (1100.65615) | K.DVLTITLTPK.V (1100.65615) |
| Peptide 3 | R.VNSAAFPAPIEK.T (1243.66811) | R.VNSAAFPAPIEK.T (1243.66811) |
